# Perioperative changes in fluid distribution and haemodynamics in acute high-risk abdominal surgery

**DOI:** 10.1186/s13054-023-04309-9

**Published:** 2023-01-16

**Authors:** Mirjana Cihoric, Henrik Kehlet, Jakob Højlund, Morten Laksáfoss Lauritsen, Katrine Kanstrup, Nicolai Bang Foss

**Affiliations:** 1 Department of Anaesthesiology and Intensive Care Medicine, Copenhagen University Hospital Hvidovre, Kettegaard Allé 30, 2650 Hvidovre, Copenhagen, Capital Region of Denmark Denmark; 2 Section for Surgical Pathophysiology, JMC, Rigshospitalet, Copenhagen, Capital Region of Denmark Denmark; 3 Gastrounit, Surgical Section, Copenhagen University Hospital Hvidovre, Hvidovre, Capital Region of Denmark Denmark

**Keywords:** Emergency laparotomy, Fluid administration, Goal-directed therapy, Haemodynamics, Overhydration, Preload dependency

## Abstract

**Background:**

Understanding the pathophysiology of fluid distribution in acute high-risk abdominal (AHA) surgery is essential in optimizing fluid management. There is currently no data on the time course and haemodynamic implications of fluid distribution in the perioperative period and the differences between the surgical pathologies.

**Methods:**

Seventy-three patients undergoing surgery for intestinal obstruction, perforated viscus, and anastomotic leakage within a well-defined perioperative regime, including intraoperative goal-directed therapy, were included in this prospective, observational study. From 0 to 120 h, we measured body fluid volumes and hydration status by bioimpedance spectroscopy (BIA), fluid balance (input vs. output), preload dependency defined as a > 10% increase in stroke volume after preoperative fluid challenge, and post-operatively evaluated by passive leg raise.

**Results:**

We observed a progressive increase in fluid balance and extracellular volume throughout the study, irrespective of surgical diagnosis. BIA measured variables indicated post-operative overhydration in 36% of the patients, increasing to 50% on the 5th post-operative day, coinciding with a progressive increase of preload dependency, from 12% immediately post-operatively to 58% on the 5th post-operative day and irrespective of surgical diagnosis. Patients with overhydration were less haemodynamically stable than those with normo- or dehydration.

**Conclusion:**

Despite increased fluid balance and extracellular volumes, preload dependency increased progressively during the post-operative period. Our observations indicate a post-operative physiological incoherence between changes in the extracellular volume compartment and inadequate physiological preload control in patients undergoing AHA surgery. Considering the increasing overhydration during the observational period, our findings show that an indiscriminate correction of preload dependency with intravenous fluid bolus could lead to overhydration.

*Trial registration* clinicaltrials.gov. (NCT03997721), Registered 23 May 2019, first participant enrolled 01 June 2019.

**Supplementary Information:**

The online version contains supplementary material available at 10.1186/s13054-023-04309-9.

## Introduction

Inadequate fluid management after initial haemodynamic resuscitation can have detrimental consequences [1]. Several studies have demonstrated a positive correlation between overhydration and adverse outcomes in critically ill patients [1–3]. On the other hand, intravascular volume depletion leads to an increased risk of acute kidney injury [4]. However, there are currently no studies focusing on post-operative fluid treatment beyond the immediate post-operative period [5, 6], and the optimal therapeutic target parameters for volume control and fluid therapy after the acute stage of post-operative critical illness remain unclear.

There is some evidence [7] that the use of cardiac output monitoring or goal-directed therapy approach to guide intravenous fluid administration as part of a haemodynamic therapy algorithm modifies inflammatory pathways [8], improves tissue perfusion and oxygenation [9], and reduces post-operative complication rates and hospital stay [10] when applied to elective surgery. However, there is still an ongoing debate on whether this treatment applies to all types of surgical populations [11].

The term acute high-risk abdominal (AHA) surgery [12, 13] encompasses a surgical exploration of the acute abdomen for several underlying pathologies, with intestinal obstruction, perforation, and peritonitis being the most frequent. These patients often suffer from hypovolaemia, dehydration, and sepsis, which may result in extravascular fluid accumulation and post-operative organ dysfunctions, complicating fluid management and influencing patient outcomes [10, 14, 15]. Despite the suggestion of benefit in elective surgery, cardiac output-guided resuscitation may not be generalizable to patients undergoing AHA surgery, where similarities with critically ill patients are many and in whom the evidence for fluid resuscitation based on cardiac output is uncertain [4, 10, 16, 17].

As such, the assessment of hydration status in AHA surgery and consequent fluid treatment are still complex and require an in-depth knowledge of body fluid homeostasis to establish a strategy that optimizes tissue perfusion and identifies the transition from necessary fluid resuscitation to harmful fluid volume accumulation [18, 19]. Additionally, it is essential to consider the diversity in the pathophysiology of patients undergoing AHA surgery, as an association between overhydration and the negative outcome may be dependent on the surgical diagnosis [20]

The present study aimed to assess the perioperative fluid changes and haemodynamics in intestinal obstruction, perforated viscus, and anastomotic leakage following elective surgery within a goal-directed therapy approach.

## Methods

This was a single-centre, prospective observational cohort study from 01–06-2019 to 25–02-2021 at the department of Anaesthesiology and Intensive Care and the department of Gastrointestinal Surgery at Hvidovre University Hospital. The ethics committee approved the study (H-19010653), The Danish Data Protection Agency (VD-2019-121) and registered at clinicaltrials.gov. (NCT03997721). We followed Strengthening the Reporting of Observational Studies in Epidemiology (STROBE) guidelines.

### Patient population

After verbal and written consent, we included all adults (18 y/o or over) undergoing AHA surgery for primary intestinal obstruction (small and large intestine), perforated viscus (defined as either perforated ulcer, small or large intestine), and anastomotic leakage following elective surgery.

**Fig. 1:**
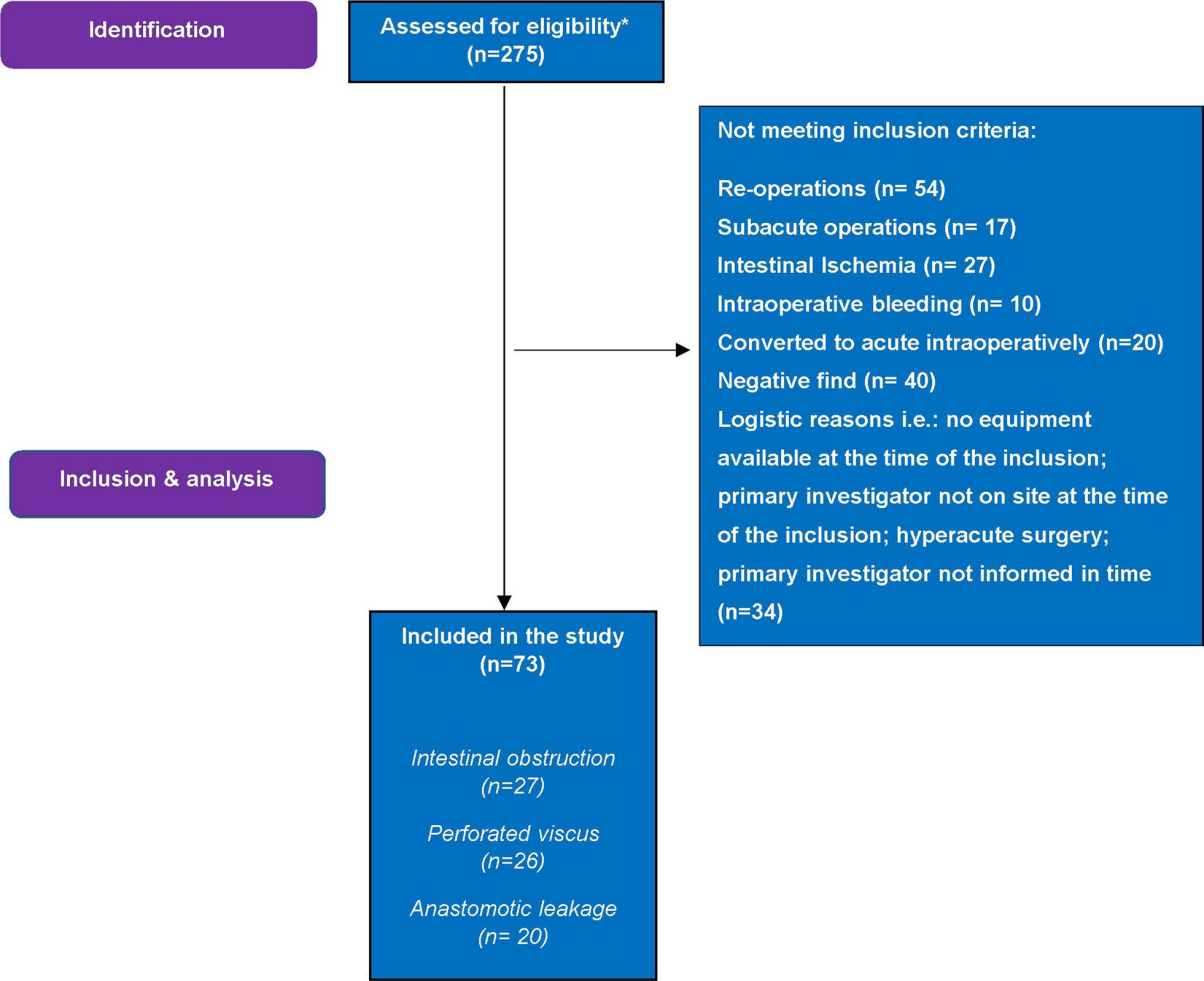
Flowchart of identification, inclusion, and exclusion criteria of patient undergoing acute high-risk abdominal surgery. *Inclusion pause due to COVID-19

Intestinal ischemia, abdominal bleeding, reoperations not including anastomotic leakage following elective surgery, and subacute surgeries (scheduled within 48H after initial diagnosis) were excluded. Elective surgeries converted intraoperatively to acute were also excluded. Patients with a negative find (no acute abdominal pathology discovered during surgery) missing data on fluid administration, vasopressor and inotropes administration, plasma electrolyte levels, and patients transferred to another hospital immediately following surgery were also excluded (Fig. 1).

### Outcome

The primary outcome was to describe the hydration status and fluid distribution (including the association with registered volume administration) measured by bioimpedance spectroscopy analysis (BIA) during the early perioperative period in patients with intestinal obstruction vs. perforated viscus vs. anastomotic leakage following elective surgery. Secondarily, we wanted to explore the association between perioperative haemodynamics, and hydration status measured by BIA.

### Perioperative management

At this surgical centre, a well-established multimodal standardized protocol is applied to patients undergoing AHA surgery [12], following recent National Emergency Laparotomy Audit guidelines [13], including haemodynamic monitoring with invasive arterial pressure intraoperatively as well as during Post-Anaesthesia Care Unit (PACU) or Intensive Care (ICU) stay, preoperative stroke volume (SV)-guided fluid and vasopressor management (LiDCOrapidTM; LiDCO, London, UK), neuraxial analgesia and anaesthesia.

### Study protocol

**Fig. 2:**
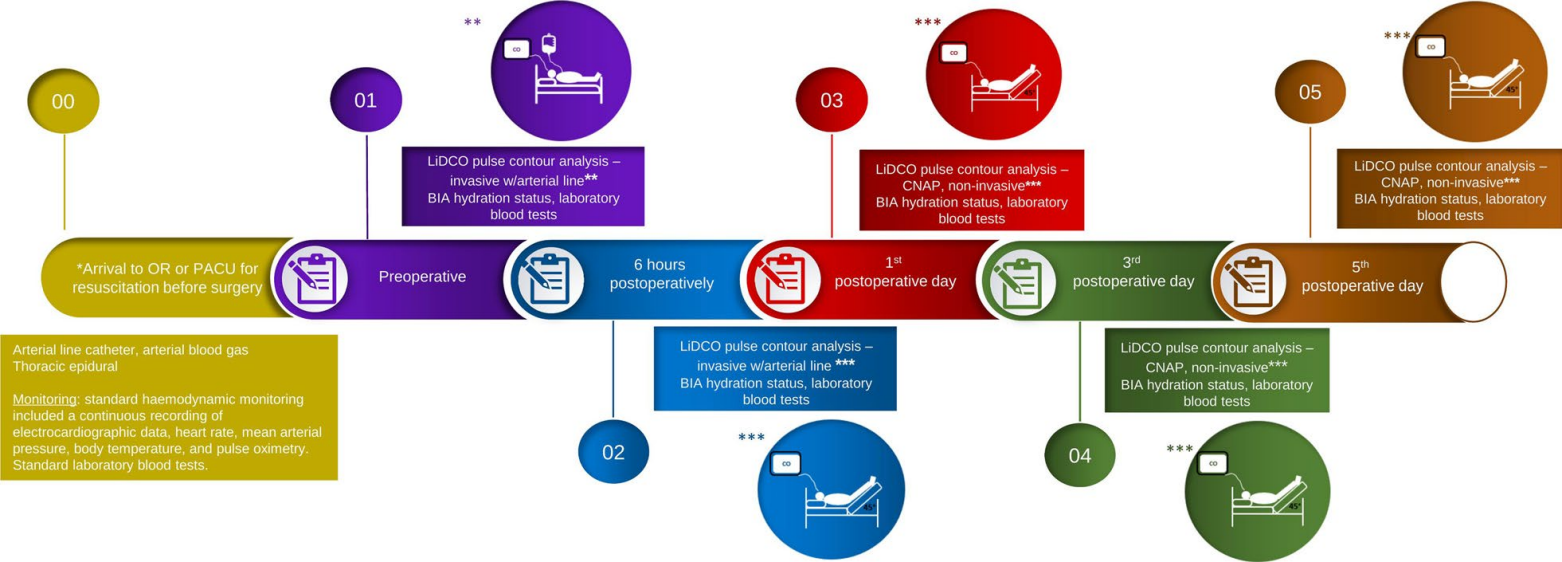
Timeline of data collection. *As per institutional protocol for acute high-risk abdominal surgery. **Fluid challenge w/250 mL human albumin; ***Passive leg raise; BIA: bioimpedance spectroscopy analysis; CNAP: The LiDCO continuous non-invasive arterial pressure haemodynamic monitoring; LiDCO: Lithium Dilution Cardiac Output haemodynamic monitoring; OR: operating room; PACU: Post-Anaesthesia Care Unit

All study-related measurements were performed by the primary study investigator, and attending physicians were unaware of the results. After initial patient triage and diagnostic workup leading to a decision to operate, the study protocol was initiated. Figure 2 summarizes the steps:

#### Bioimpedance spectroscopy analysis (BIA) hydration status and definition of overhydration

BIA assesses body composition and estimates total body water (TBW) and extracellular water (ECW) volumes based on the tissue’s capacity to conduct electrical impulses [21].

The BIA device displays absolute fluid overload/overhydration (AFO), the difference between normal, expected ECW and the measured ECW, expressed in litres, as well as relative fluid overload/overhydration (RFO), absolute fluid overload/extracellular water ratio (AFO/ECW), expressed in percentages. A negative AFO indicates underhydration, while a positive one indicates overhydration. Based on RFO values, overhydration was defined as RFO > 15% [22, 23]. BIA was assessed using the Body Composition Monitor (BCM, Fresenius Medical Care, Germany) as proposed by the manufacturer.

*Preload dependency*Preoperative preload dependency was assessed by the initial fluid challenge, performed routinely during the resuscitation phase before general anaesthesia and neuraxial analgesia, with 250 mL human albumin solution as a bolus over 5 min. Preload dependency was defined as a > 10% increase in stroke volume [24].

post-operative preload dependency was assessed by the passive leg raise manoeuvre [25], and the monitoring (when the patient was admitted to the surgical ward) was done with continuous non-invasive arterial pressure (CNAP) [26] (Non-invasive LiDCOrapidTM; LiDCO, London, UK). Passive leg raise was performed with the patients placed in a semi-recumbent position. The trunk was then lowered to a supine position, while the legs were elevated up to 45 degrees. After 2 min, the patients were placed back in a semi-recumbent position.

Stroke volume-guided fluid management or passive leg raise measurements were not a part of standard post-operative routine and were regarded purely as a research parameter. No treating physician or nurse were at any time aware of the results, thereby not influencing the post-operative fluid management. It is important to underline that no fluid was administered as a direct consequence of the result of the PLR manoeuvre. Post-operative fluid administration was done at the discretion of the treating physician with no interference from the primary investigator.

#### Fluid balance

Daily fluid balance was defined as the difference between total input (all fluids, nutrition, blood products, medications) and total output (losses through urinary, gastrointestinal, or other drainage tubes), not including insensible losses. Cumulated fluid balance from 06.00 to 06.00 the following morning was calculated as the algebraic sum of daily fluid balance during the first five post-operative days. Fluid balance was determined from six o’clock in the morning from the previous day.

### Exposures

Haemodynamic and bioimpedance variables were collected at; baseline (before surgery) and 6 h after surgery. The same procedure was applied on the 1st, 3rd, and 5th post-operative day.

The following variables were recorded upon inclusion: demographic, clinical history (comorbidities), American Society of Anaesthesiology classification, ECOG performance score [27], Carlson comorbidity score, and qSOFA from electronic patient records. Further data collection included perioperative fluid and vasopressor administration, weight, perioperative haemodynamic parameters (SV, CO, HR, MAP); bioimpedance spectroscopy variables (AFO, RFO, BIA FO, TBW, Intracellular water (ICW), ECW), plasma lactate, C-reactive protein, sodium, potassium, albumin, and pro-brain natriuretic peptide (BNP). Body temperature, urine output, Mannheim peritonitis index (MPI) [28], use of epidural analgesia, length of hospital stay, ICU admission. Thirty-day major post-operative complications were registered according to Clavien–Dindo classification [29], specifically: pulmonary (pulmonary oedema, ultrasound-guided pleural drainage, admission to the ICU due to respiratory failure), gastrointestinal (emergency reoperations for intestinal obstruction, perforated viscus, anastomotic leakage or surgical wound infection, emergency endoscopy for bleeding ulcer, ultrasound-guided drainage of intraabdominal abscess, ICU stay due to septic shock) and renal (acute kidney injury, need for renal replacement therapy) where we applied Rifle criteria for acute kidney disease [30].

## Statistical analysis

Based on a recent study [20], we estimated an incidence of patients with overhydration on the 5th post-operative day at 50%. To estimate the assumed incidence and intending an equal inclusion, with the confidence interval (38–62), we needed to include at least 70 patients.

Data are presented by descriptive statistics (nonparametric distribution: medians with 25th–75th inter-quartile ranges (IQR) and range, normal distribution means with 95% CI and range). Normal distribution was assessed from Q–Q plots and histograms and Kolmogorov–Smirnoff's test. Categorical data were analysed using the chi-square test. Continuous data were analysed with Kruskal–Wallis test. The unpaired t test or Mann–Whitney test was used to compare data between the groups. Univariate analysis for association with outcome was applied.

All statistical assessments were done by a two-sided test using a *p* value at a 0.05 level of significance. All analyses were performed using R statistical software. (www.r-project.org).

## Results

From 01-06-2019 to 25-02-2021, 275 patients were assessed for eligibility, and 202 patients did not meet the inclusion criteria, where 34 were not included due to logistic reasons (detailed description: Fig. 1). There was a pause in inclusion due to COVID-19 pandemic—from March 2020–September 2020. Seventy-three patients were included in the study; 27 underwent AHA surgery for intestinal obstruction, 26 for perforated viscus, and 20 for anastomotic leakage. Data entry was complete for the cohort with no variables exceeding 10% of missing data.

Descriptive data are shown in Table 1. Median (IQR) values for 5-day cumulative fluid balance were 3.4 L (1.1–11.4), 6.9 L (3.0–11.5), and 1.3 L (− 3.1–6.8) for intestinal obstruction, perforated viscus, and anastomotic leakage, respectively (*p* = 0.014) (Table 1). During the first 5 days after surgery, patients received crystalloids primarily, with cumulated 5-day administration highest in patients with anastomotic leakage 8.6 L (5.7–11.2), compared to intestinal obstruction, 6.6 L (4.5–10.1) and perforated viscus, 5.1 L (4.2–9.9), though the difference was not statistically significant.

**Table 1:** Descriptive statistics

Variables	Intestinal obstruction (*n* = 27)	Perforated viscus (*n* = 26)	Anastomotic leakage (*n* = 20)	*P* value (chi-square)
*Baseline, n (%)*				
Age, years, range	64 (27–90)	67.5 (22–87)	60 (25–80)	0.598†
Female	13 (48.1)	13 (50.0)	8 (40.0)	0.780
BMI, kg/m^2^, median (IQR)	25 (22–32)	26 (20–30)	28 (22–32)	0.239
ASA score > II	10 (37.3)	11 (42.3)	6 (30.0)	0.692
ECOG performance score > 1	12 (44.5)	14 (53.8)	6 (30.0)	0.270
Charlson Comorbidity Index ≥ 3	16 (59.2)	14 (53.8)	12 (60.0)	0.893
qSOFA, > 1	1 (3.7)	3 (11.5)	7(35.0)	0.010
*Comorbidities, n (%)*				
Cardiovascular	7 (25.9)	9 (34.6)	5 (25.0)	0.712
Ischemic heart disease	1 (3.7)	2 (7.7)	0 (0.0)	0.710
Congestive heart failure	1 (3.7)	2 (7.7)	1 (5.0)	0.700
Pulmonary	4 (14.8)	3 (11.5)	2 (10.0)	0.811
Cerebrovascular	2 (7.4)	2 (7.7)	0 (0.0)	0.449
Renal	1 (3.7)	3 (11.5)	2 (10.0)	0.551
Diuretics	3 (11.1)	5 (19.2)	4 (20.0)	0.284
*Preoperative blood samples, median (IQR)*				
Plasma albumin, g/L	35 (28–41)	30 (25–36)	26 (22–27)	< .001
C-reactive protein, mg/dL	10 (5–10)	70 (10–210)	320 (250–355)	< .0001
Creatinine, mmol/L	78 (62–92)	82 (68–124)	87 (68–94)	0.456
Plasma sodium, mmol/L	137 (134–139)	136 (135–139)	136 (134–139)	0.052
Plasma potassium, mmol/L	3.9 (3.6–4.0)	3.6 (3.5–4.2)	3.8 (3.6–4.1)	0.165
Plasma chloride, mmol/L	102 (99–106)	106 (102–108)	104 (101–107)	0.257
Plasma lactate, mmol/L	0.8 (0.7–1.8)	1.2 (0.8–1.9)	1.1 (0.8–1.7)	0.379
*Intraoperative parameters, n (%)*				
Laparoscopy	2 (7.4)	8 (30.8)	7 (35.0)	0.029
Laparotomy	25 (92.6)	18 (69.2)	13 (65.0)	0.029
Epidural analgesia	20 (74.1)	24 (92	18 890.0)	0.199
Vasopressor infusion*	14 (51.8)	18 (69.2)	17 (85.0)	0.050
Manheim Peritonitis Index > 20	7 (26.0)	14 (53.8)	8 (40.0)	0.116
*Volume Status Evaluation, median (IQR)*				
Intraoperative fluid balance, *L*	1.8 (1.4–2.1)	1.9 (1.5–2.6)	1.6 (1.2–2.2)	0.340¤
Crystalloids, L	1.0 (0.7–1.9)	1.3 (1.0–1.6)	1.1 (0.8–1.3)	0.352¤
Colloids, L	0.5(0.4–0.8)	0.8 (0.5–1.3)	0.5 (0.4–0.8)	0.230¤
Cumulated I/O, L, days 1–5	3.4 (1.1–11.4)	6.9 (3.0–11.5)	1.3 (− 3.1–6.8)	0.014¤
Cumulated crystalloids, L, days 1–5	5.1 (4.2–9.9)	6.6 (4.5–10.1)	8.6 (5.7–11.2)	0.200¤
Cumulated colloids, L, days 1–5	0.0 (0.0–0.8)	0.3 (0.0–0.7)	0.1 (0.0–0.5)	0.610¤
Body weight change, kg: days 1–5	2.7 (− 1.5–6.0)	4.5 (1.0–8.1)	1.2 (− 1.2–2.3)	0.152¤
AFO volume change, L: days 1–5	3.35 (1.40–5.20)	5.15 (3.90–5.80)	3.50 (1.75–4.50)	0.044¤

*BMI* Body Mass Index; *ASA* American Society of Anaesthesiologists; *ECOG* Eastern Cooperative Oncology Group performance score; *qSOFA* quick Sepsis Related Organ Failure Assessment; Colloids = albumin 5%; I/O: fluid balance calculated by the volume of fluid intake minus the volume of fluid output in the defined duration of time; *AFO* absolute fluid overload measured by bioimpedance spectroscopy

*Phenylephrine or Norepinephrine

†One way ANOVA

¤Kruskal–Wallis

**Fig. 3:**
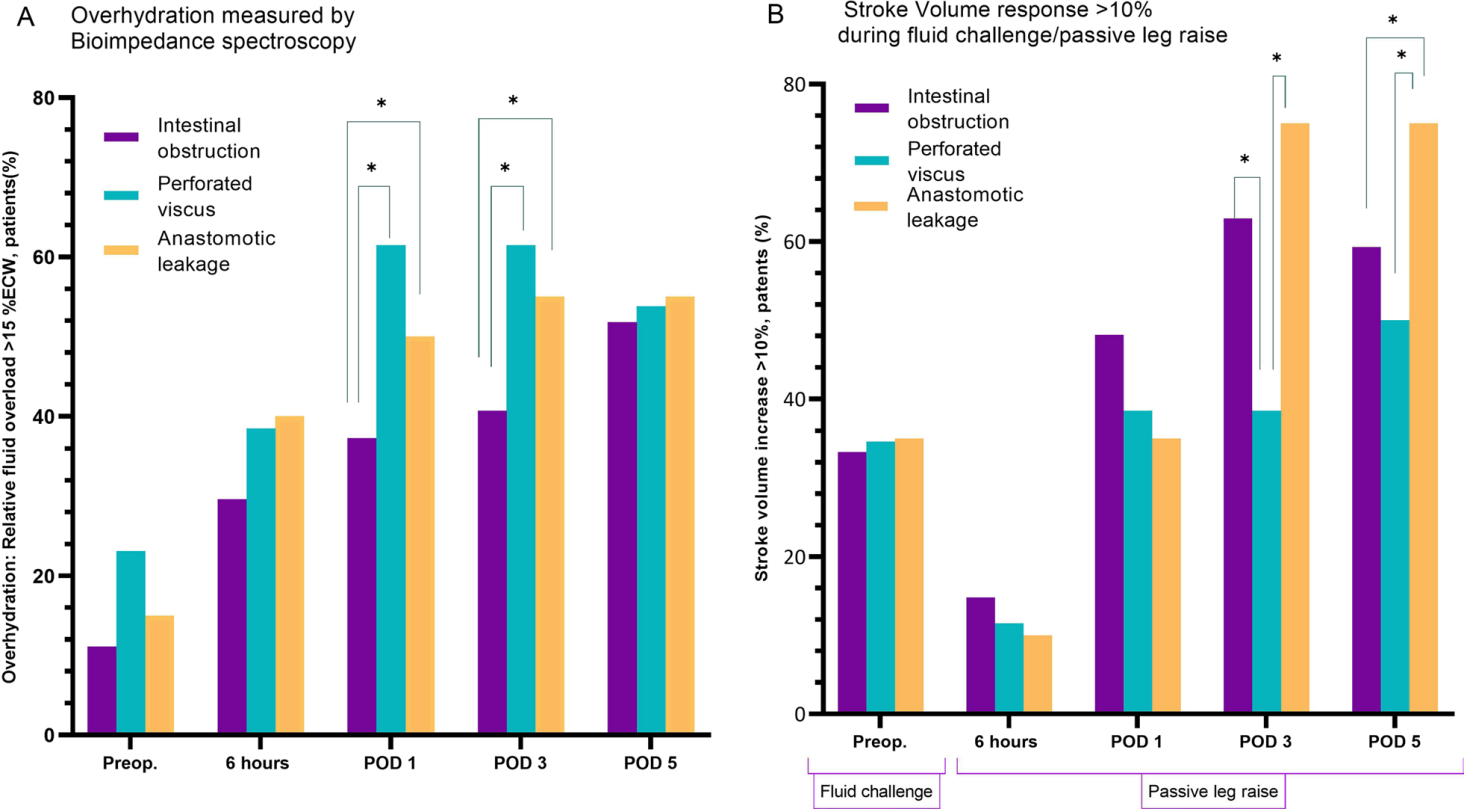
Preload dependency and overhydration in the perioperative period for acute high-risk abdominal surgery: **A** Overhydration, defined as relative fluid overload (RFO) > 15%, measured by bioimpedance spectroscopy; **B** preload dependency defined as stroke volume increase during fluid challenge or passive leg raise manoeuvre, *p < *0.05*

Before surgery, 16% of the population had BIA measured overhydration (RFO > 15%) (Fig. 3A), and the majority of these were patients had perforated viscus. By the 5th post-operative day, 50% of all patients were overhydrated, with no statistical difference between groups.

Table 2 shows intraoperative fluid administration and changes in volume status. Six hours after surgery, the number of patients with BIA measured overhydration was 26 (36%), significantly higher (*p* < 0.001) than before surgery. This was consistent for all 3 groups (*p* < 0.01, *p* < 0.001, *p* = 0.024, respectively). Absolute overhydration increased significantly by 1.77 ± 1.4 L (*p* < 0.0001), from 0.68 ± 2.5 L preoperatively to 2.4 L ± 2.7 post-operatively, equivalent to a significant 10% rise in ECW. Total body water and intracellular water increased without reaching statistical significance (*p* = 0.430 and 0.876, respectively).

**Table 2:** Bioimpedance volume status assessment in the pre- and post-surgery period

Variables	Preoperative	6 h post-operative	Mean difference	*P* value (Wilcox)
*All patients*				
TBW, L	39.8 ± 8.1	41.0 ± 8.2	0.1 ± 4.9	0.430
ICW, L	21.4 ± 5.0	21.4 ± 5.1	− 1.0 ± 2.5	0.876
ECW, L	18.3 ± 3.8	19.8 ± 4.0	1.5 ± 1.7	0.022
ECW/ICW ratio	0.87 ± 0.2	0.96 ± 0.2	–	
AFO, L	0.68 ± 2.5	2.4 ± 2.7	1.77 ± 1.4	< .0001
RFO, median (IQR), %	3.1 (− 5.7–11.7)	9.5 (4.3–22.2)	–	0.0001
Patients with overhydration, *n* (%)	12 (16.4)	26 (36.0)	–	< .0001**
*Intestinal obstruction patients*				
TBW, L	40.1 ± 7.8	40.5 ± 7.9	0.4 ± 2.5	0.802
ICW, L	22.2 ± 4.8	21.2 ± 5.0	1.0 ± 1.7	0.287
ECW, L	17.9 ± 3.5	19.1 ± 3.5	1.2 ± 1.8	0.197
ECW/ICW ratio	0.82 ± 0.1	0.92 ± 0.2	–	
AFO, L	− 0.2 ± 2.2	1.9 ± 2.6	2.0 ± 1.8	0.003
RFO, median (IQR), %	− 0.5 (− 9.0–7.8)	7.7 (0.5–16.7)	–	0.003
Patients with overhydration, *n* (%)	3 (11.1)	8 (29.6)	–	0.004**
*Perforated viscus patients*				
TBW, L	38.7 ± 9.1	41.4 ± 8.7	2.7 ± 4.2	0.308
ICW, L	20.3 ± 5.9	21.6 ± 5.8	1.2 ± 2.7	0.404
ECW, L	18.2 ± 4.1	20.0 ± 6.7	1.8 ± 2.0	0.107
ECW/ICW ratio	0.93 ± 0.2	0.96 ± 0.2	–	
AFO, L	1.0 ± 2.8	2.5 ± 3.0	1.5 ± 1.3	0.046
RFO, median (IQR), %	5.1 (− 3.0–13.8)	10.9 (2.4–23.7)	–	0.035
Patients with overhydration, *n* (%)	6 (23.1)	10 (38.5)	–	0.0004**
*Anastomotic leakage patients*				
TBW, L	40.7 ± 7.3	40.7 ± 8.3	0.0 ± 7.0	0.241
ICW, L	21.7 ± 4.0	21.4 ± 4.5	− 0.3 ± 2.7	0.247
ECW, L	19.0 ± 3.9	20.4 ± 3.4	1.4 ± 1.2	0.261
ECW/ICW ratio	0.88 ± 0.1	1.0 ± 0.2	–	
AFO, L	1.5 ± 2.1	3.1 ± 1.9	2.0 ± 1.0	0.083
RFO, median (IQR), %	5.8 (− 2.0–14.1)	11.9 (8.5–22.8)	–	0.011
Patients with overhydration, *n* (%)	3 (15.0)	8 (40.0)	–	0.024**

Data are expressed as mean ± SD unless otherwise stated

*AFO* absolute fluid overload, *ECW* extracellular water, *ICW* intracellular water, *RFO* relative fluid overload/overhydration (percentage of ECW), *TBW* total body water; overhydration defined as RFO > 15% of ECW

**Chi-square

**Fig. 4:**
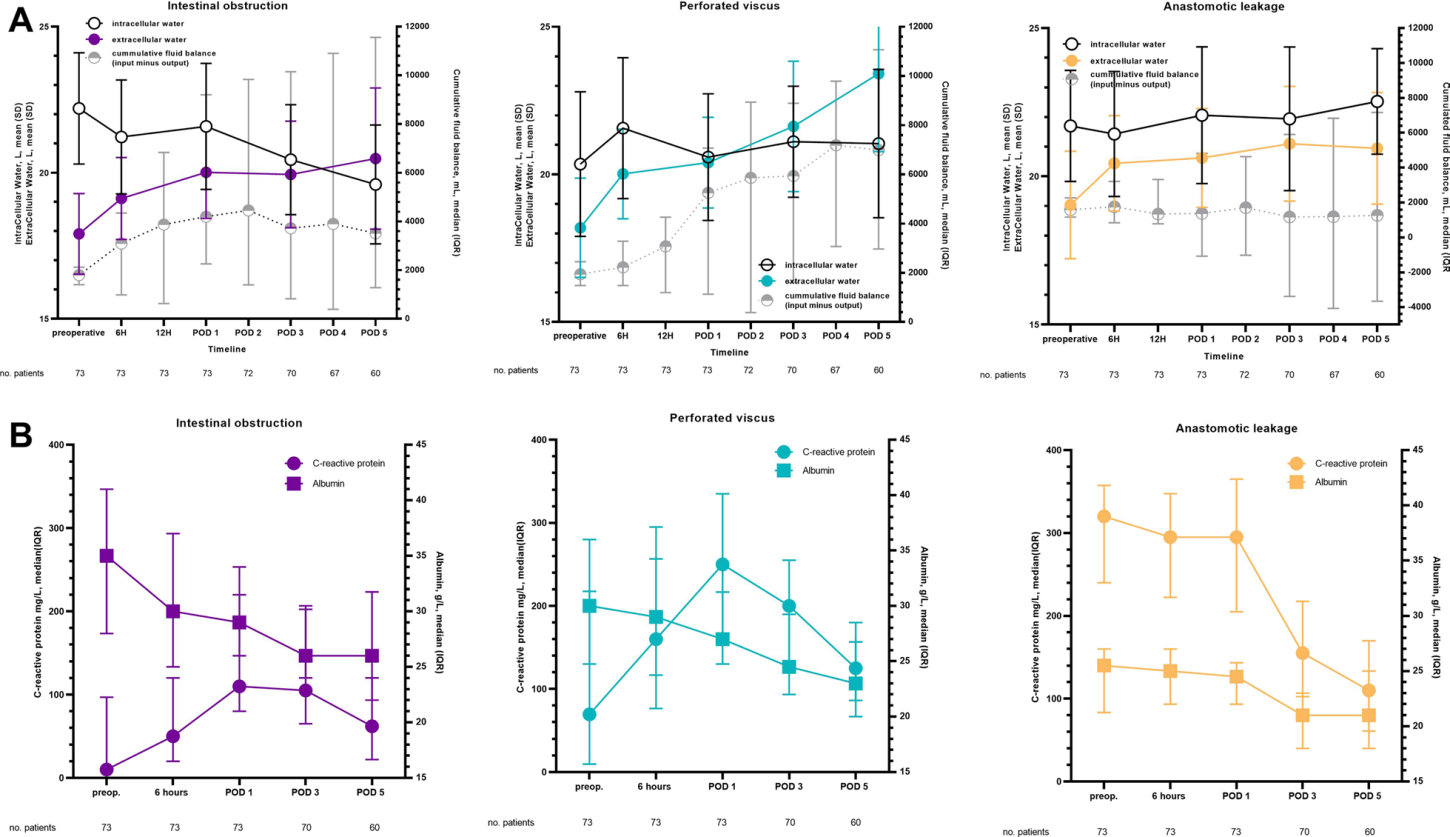
Dynamic changes between compartments in the perioperative period and inflammatory markers for acute high-risk abdominal surgery. **A** relationship between registered cumulative fluid balance and BIA measured fluid shifts and **B** inflammatory markers during the perioperative period in acute high-risk abdominal surgery

Figure 4 shows absolute changes in fluid compartments and inflammatory markers throughout the perioperative period. Extracellular water increased across the cohort, most pronounced in patients with perforated viscus (ΔECW 5.91 ± 5.83L). Intracellular water decreased in patients with intestinal obstruction (ΔICW − 2.15 ± 2.71), while it remained unchanged or slightly increased throughout the perioperative course for patients with perforated viscus and anastomotic leakage (ΔICW 0.66 ± 2.62L and ΔICW 1.79 ± 4.30L, respectively). A post-operative fall in plasma albumin occurred in the whole cohort, although most profound in IO, Δalbumin -6.7 ± 5.5 g/L, *p* = 0.001. Inflammatory response peaked at 1st POD in both intestinal obstruction and perforated viscus IO and PV, with ΔCRP increase 80 ± 104 mmol/L and ΔCRP 119 ± 119 mmol/L (*p* < 0.001), respectively. The C-reactive protein remained unchanged (ΔCRP 1 ± 104 mmol/L) in patients with anastomotic leakage, beginning a downward trajectory on 3rd POD (Fig. 4).

Preoperative preload response to a fluid bolus was seen in 34% of all patients, with no statistical differences between groups (Fig. 3B). There was a significant reduction of preload dependency 6 h after surgery (12%), but a progressive post-operative preload dependency assessed by PLR increased progressively throughout the cohort, peaking on the 5th post-operative day. Thus, 75% of the patients with AL responded to passive leg raise, significantly higher than IO (59%) and PV (50%). Before surgery, 50% of the patients with overhydration were preload dependent (Additional file 1: Appendix 1), and the number increased throughout the observational period, irrespective of hydration status.

Patients with overhydration were less haemodynamically stable, with significantly higher cardiac biomarker proBNP during the entire observational period as well as incidence of septic shock, with no difference in preoperative cardiac pathology (Additional file 2: Appendix 2).

The median length of epidural analgesia in our cohort was 3 days, and 70% of the patients had their epidural discontinued by day 3. We found no difference in the incidence of preload dependence when stratified according to the presence of epidural analgesia (Additional file 3: Appendix 3).

The incidence of post-operative major complications including death was significantly higher in patients with perforated viscus (65%), than in patients with intestinal obstruction (26%) and anastomotic leakage (35%), *p* = 0.003 (Table 3), as was the need for ICU admission immediately after surgery with *p* = 0.026. Although there was no statistically significant difference in the overall incidence of pulmonary complications between groups (*p* = 0.208), patients with perforated viscus had a significantly higher need for ultrasound-guided pleural drainage (39%) than intestinal obstruction (4%) and anastomotic leakage (10%), *p* = 0.048. Also, 35% of the patients with anastomotic leakage were admitted to the ICU due to respiratory failure, compared to intestinal obstruction (4%) and perforated viscus (15%), without reaching statistical significance (*p* = 0.105).

**Table 3:** Post-operative outcomes in acute high-risk abdominal surgery

*N* (%)	Intestinal obstruction (*n* = 27)	Perforated viscus (*n* = 26)	Anastomotic leakage (*n* = 20)	*P* value (chi-square)
30-day mortality	1 (3.7)	3 (11.5)	2 (10.0)	0.834
Patients with at least one 30-day major complication (CD > II)	7 (25.9)	17 (65.4)	7 (35.0)	0.003
ICU admission immediately after surgery	3 (11.1)	11 (42.3)	4 (20.0)	0.026
Need for respiratory support*	1 (3.7)	6 (23.1)	1 (5.0)	0.070
Need for vasopressors beyond 24 h after surgery*	6 (22.2)	10 (38.4)	2 (10.0)	0.079
*Renal*				
Acute kidney injury**	4 (14.8)	10 (38.5)	3 (15.0)	0.074
Need for post-operative RTT	0 (0.0)	4 (15.4)	3 (13.4)	0.059
Gastrointestinal	6(22.2)	14 (53.8)	8 (40.0)	0.011
Reoperation for acute abdominal pathology	4 (14.8)	9 (34.6)	7(35.0)	0.245
Reoperation for surgical wound infection	1 (3.7)	4 (15.4)	0 (0.0)	0.271
US-guided drainage of intraabdominal abscess	4 (14.8)	2 (7.6)	1 (5.0)	0.649
Admission to ICU* due to postop. septic shock	2 (7.4)	9 (34.6)	5 (25.0)	0.499
Pulmonary	7 (26.0)	14 (53.8)	8 (40.0)	0.208
Pulmonary oedema	5 (18.5)	6 (23.1)	3 (15.0)	0.146
US-guided pleural drainage	1 (3.7)	10 (38.5)	2 (10.0)	0.048
Admission to ICU* due to respiratory failure	1 (3.7)	4 (15.4)	7 (35.0)	0.105

*CD* Clavien–Dindo criteria for post-operative complications; *ICU* Intensive Care Unit; *RRT* renal replacement therapy; * emergency reoperations for intestinal obstruction, perforated viscus, anastomotic leakage or surgical wound infection; *US* ultrasound-guided drainage of intraabdominal abscess, *ICU* stay due to septic shock; Pulmonary: *US* ultrasound-guided pleural drainage, admission to ICU due to respiratory failure, X-ray-verified pulmonary oedema

*At any point during the initial hospital stay

**RIFLE: criteria for acute kidney injury

## Discussion

During the first five days after AHA surgery, we found progressive overhydration, measured by bioimpedance spectroscopy, irrespective of surgical diagnosis. Simultaneously, we observed a progressive increase in preload dependency, as evaluated by passive leg raise, again irrespective of surgical diagnosis.

Overhydration was most prevalent in patients with perforated viscus compared to intestinal obstruction and anastomotic leakage, with a persistent increase in extracellular volume coinciding with fluid administration. The increase in extracellular volume was present irrespective of diagnosis, although most pronounced in patients with perforated viscus. Interestingly, intracellular volume decreased for patients with intestinal obstruction but remained unchanged or slightly increased for perforated viscus and anastomotic leakage.

There is extensive literature on the perioperative fluid status and the impact of overhydration on mortality in critically ill patients [1–3]. However, limited data exist [17] on the perioperative fluid status beyond the immediate post-operative period in patients undergoing AHA surgery. These patients, with a high degree of acute inflammation, sepsis, and fluid disturbances, established even before surgery, share similarities with critically ill patients.

Since patients undergoing AHA surgery may have prolonged derangement of cardiovascular, pulmonary, and gastrointestinal function for days to weeks after primary surgery, judicious fluid replacement is needed to prevent multi-organ failure. In this study, we found that patients with overhydration were less haemodynamically stable, with significantly higher cardiac biomarker proBNP as well as incidence of septic shock, with no difference in preoperative cardiac pathology (Additional file 2: Appendix 2). As such, the haemodynamic instability could indicate either cardiac failure, septic shock, or both. Previously, several studies have found association between elevated proBNP and inflammation, and as such the causality is elusive [31, 32].

There is a consensus that applying GDT in managing perioperative fluid administration in elective surgery could reduce post-operative complications [7, 33]. In contrast, there are conflicting results regarding critical care patients [10, 16, 17], though they do have one thing in common: they focus on the immediate perioperative period with no current data on the potential application of GDT principles to guide fluid therapy beyond this period.

However, while the importance of correct late fluid management cannot be overstated [3, 34], the strategies and monitoring needed are unknown and more complex than elective surgery [35]. Studies in critically ill patients indicate that overhydration is not a problem confined to the early period. Resuscitation fluids comprise less than 10% of overall fluid intake during a whole ICU stay, about 25% are maintenance/replacement fluids, and nearly one-third of the fluid intake consists of “hidden” fluids associated with drug administration, etc. [36].

We found 16% of all patients undergoing AHA surgery to be significantly overhydrated before the application of a GDT protocol, but 50% of those still responded with a significant increase in SV during the preoperative fluid challenge. There seems to be a physiological incoherence between overhydration and preload dependency throughout the observational period, suggesting vasoplegia and endothelial dysfunction rather than absolute intravascular hypovolaemia as a driver of preload dependency.

The effect of the dyshydration might be diagnosis-specific, with patients with IO presenting with a higher degree of post-operative fluid shifts (Fig. 4). Sepsis-induced vasoplegia and endothelial dysfunction are the expected primary drivers of an increase in ECW [37], whereas loss of ICW is relatively unexplored.

Although patients with IO presenting with overhydration did have a higher degree of inflammation, as expected [20], they suffer from water depletion, resulting in hypertonicity in the extracellular space and leading to intracellular dehydration [38] and secondary protein loss [39]. This could explain the rather steep curve of protein loss in patients with intestinal obstruction compared to patients with perforated viscus and anastomotic leakage.

General and neuraxial (epidural) anaesthesia suppress the sympathetic tone, reducing preload and afterload, potentially inducing or amplifying preload dependency. The effect of an epidural is greatest in the initial post-operative period, where we found no or very low incidence of preload dependency (Fig. 3).

Still, post-operative vasoplegia due to continuous neuraxial blockage and opioid therapy, combined with post-operative inflammatory response, should be considered when assessing preload dependency in a surgical ward. Considering the increasing overhydration during the observational period, our findings show that if we were to apply correction of preload dependency with a fluid bolus as a primary basis for post-operative fluid therapy, as practiced in GDT protocols, we might create unnecessary overhydration.

The inflammatory response was on the downward trajectory by 5th POD (Fig. 4), but there was still progressive increase in preload dependence and overhydration. However, C-reactive protein did not reach baseline levels in patients with intestinal obstruction and perforated viscus and was still at a median value > 100 mg/L in patients with anastomotic leakage. Simultaneously, we observed continuous decline in plasma albumin, indicating that the inflammatory reaction is still present. These findings suggest a need for more extended studies to better understand the trajectory of preload dependency and overhydration and to determine when they start to decline.

During the post-operative period, preload dependency was assessed by passive leg raise, which is a way of challenging preload without administering fluid and thereby avoiding unnecessary fluid administration, provided cardiac output monitoring [40]. Several studies have confirmed the reliability of the passive leg raise with exceptional consistency, and passive leg raise is frequently applied in the ICU departments [40]. A recent meta-analysis found a pooled sensitivity of 85% and a pooled specificity of 91% for detecting fluid responsiveness. We measured stroke volume before and after passive leg raise when the patient has been moved back to the semi-recumbent position to check that it returns to its baseline (data not shown).

Our study has several strengths. This was the first study to assess the fluid status and fluid shifts in patients undergoing AHA surgery, a group equated to the international term emergency laparotomy, beyond the immediate intra- and post-operative period while considering the fundamental pathophysiological differences of diagnosis AHA surgery. This study suggests that specific fluid resuscitation strategies should depend on the diagnosis and highlight the discussion about the place of vasoconstriction therapy in the context of GDT protocols [41, 42].

Limitations of the study include a single-centre study and thus prone to inclusion bias. However, it was a prospective study, and the patient enrolment was unselected. Our results indicate a significant variation in fluid administration, compared to elective surgery, and a considerable amount of fluid is administered. However, there were no data on the indications for post-operative fluid management, which would have been important.

Bioimpedance spectroscopy fluid analysis has been validated in several studies evaluating different patient populations, both elective and emergent [23, 43, 44]. This analysis attempts to measure intra- and extracellular fluid volume and provides absolute and relative fluid overload, but it can be affected by absolute sodium content and thereby overestimate the volume. However, a recent study did find a correlation between the absolute fluid overload measured by bioimpedance spectroscopy and registered weight changes and fluid balance [45].

In conclusion, despite progressive overhydration throughout the perioperative period, post-operative preload dependency assessed by PLR increased steadily in patients undergoing AHA surgery, indicating a physiological incoherence between fluid status and preload dependence, where patients appeared to be volume deficient but still overhydrated. Considering the increasing overhydration during the observational period, our findings show that an indiscriminate correction of preload dependency with intravenous fluid bolus could lead to overhydration.

## Supplementary Information

**Additional file 1**. **Appendix 1:** Preload dependency and hydration status in acute high-risk abdominal surgery. RFO: relative fluid overload (measured by Bioimpedance spectroscopy)—the absolute fluid overload/extracellular water ratio (AFO/ECW), expressed in percentages; normo- or dehydration: RFO < 15%; overhydration: RFO > 15%; Preload dependency defined as stroke volume increase during fluid challenge or passive leg raise manoeuvre.

**Additional file 2**. **Appendix 2:** Changes in haemodynamic variables during the observational period stratified according to hydration status. RFO: relative fluid overload (measured by Bioimpedance spectroscopy)—the absolute fluid overload / extracellular water ratio (AFO/ECW), expressed in percentages; normo- or dehydration: RFO < 15%; overhydration: RFO > 15%; POD: post-operative day; *p<0.05.

**Additional file 3**. **Appendix 3:** Postoperative preload dependency stratified according to the days with epidural analgesia.

## Data Availability

All authors had full access to all data in the study and take responsibility for the integrity of the data. The data that support the findings of this study are available from the corresponding author, MC, upon reasonable request.

## Abbreviations

AFO: Absolute fluid overload = overhydration (L)
AHA: Acute high-risk abdominal surgery
AL: Anastomotic leakage
CNAP: Continuous non-invasive arterial pressure
CO: Cardiac output
ECOG: Eastern Cooperative Oncology Group
ECW: Extracellular water
ICU: Intensive care unit
ICW: Intracellular Water
IO: Intestinal obstruction
LiDCO: Lithium dilution cardiac output measurement
MPI: Mannheim peritonitis index
NELA: National Emergency Laparotomy Audit
PACU: Post-Anaesthesia Care Unit
PLR: Passive leg raise
PV: Perforated viscus
RFO: Relative fluid overload = overhydration (% ECW)
SV: Stroke volume
TBW: Total body water
