# Supplementary figures and images for: Perioperative changes in fluid distribution and haemodynamics in acute high-risk abdominal surgery
Source: Crit Care. 2023 Jan 16;27:20. doi: 10.1186/s13054-023-04309-9 (PMC9841944; doi:10.1186/s13054-023-04309-9)

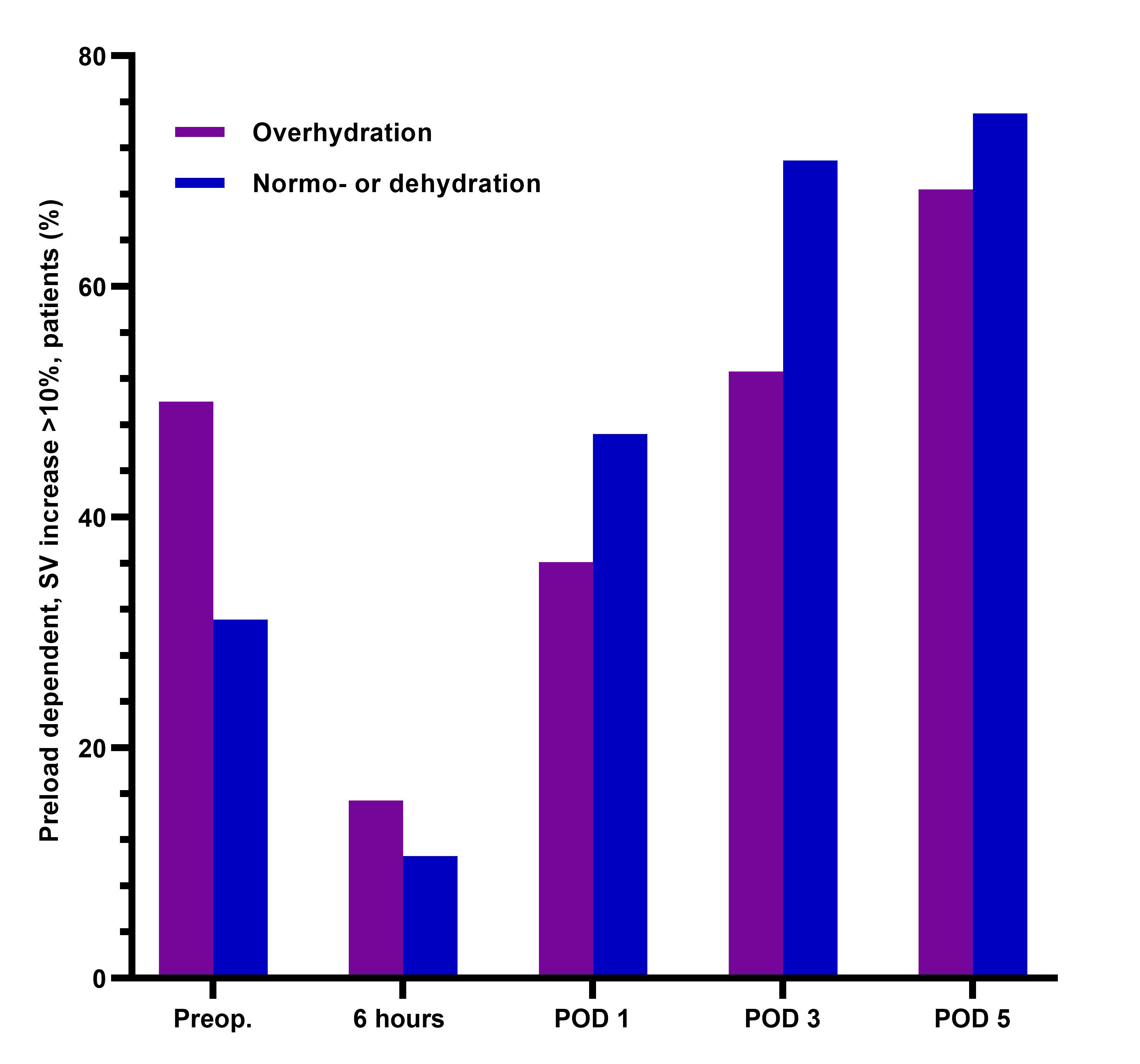

Supplement: Supplementary file 1 — Additional file 1. Appendix 1: Preload dependency and hydration status in acute high-risk abdominal surgery. RFO: relative fluid overload (measured by Bioimpedance spectroscopy)—the absolute fluid overload/extracellular water ratio (AFO/ECW), expressed in percentages; normo- or dehydration: RFO < 15%; overhydration: RFO > 15%; Preload dependency defined as stroke volume increase during fluid challenge or passive leg raise manoeuvre. [file 13054_2023_4309_MOESM1_ESM.jpg]

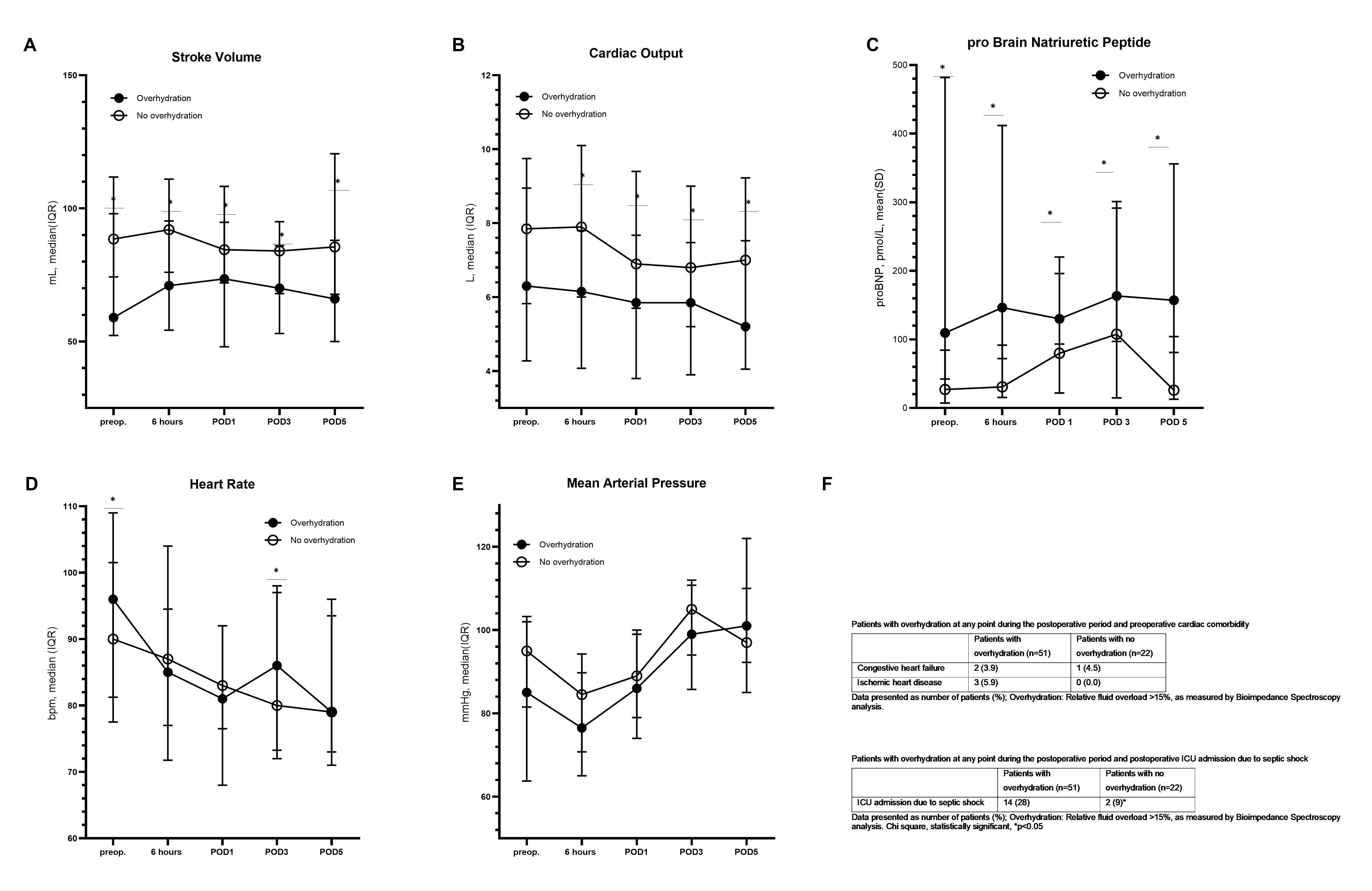

Supplement: Supplementary file 2 — Additional file 2. Appendix 2: Changes in haemodynamic variables during the observational period stratified according to hydration status. RFO: relative fluid overload (measured by Bioimpedance spectroscopy)—the absolute fluid overload / extracellular water ratio (AFO/ECW), expressed in percentages; normo- or dehydration: RFO < 15%; overhydration: RFO > 15%; POD: post-operative day; *p<0.05. [file 13054_2023_4309_MOESM2_ESM.jpg]
