# Supplementary material for: Perioperative changes in fluid distribution and haemodynamics in acute high-risk abdominal surgery
Source: Crit Care. 2023 Jan 16;27:20. doi: 10.1186/s13054-023-04309-9 (PMC9841944; doi:10.1186/s13054-023-04309-9)
Supplement: Supplementary file 3 — Additional file 3. Appendix 3: Postoperative preload dependency stratified according to the days with epidural analgesia. [file 13054_2023_4309_MOESM3_ESM.docx]

Appendix 3:

Postoperative preload dependency stratified according to the days with epidural analgesia

| **6 hours after surgery** | Epidural Analgesia  (n=62) | No Epidural Analgesia  (n=11) |
| --- | --- | --- |
|  |  |  |
| Preload dependency | 8 (12) | 1 (9) |

| **1^st^ postoperative day** | Epidural Analgesia  (n=63) | No Epidural Analgesia  (n=10) |
| --- | --- | --- |
|  |  |  |
| Preload dependency | 26 (4) | 4 (40) |

| **3^rd^ postoperative day** | Epidural Analgesia  (n=42) | No Epidural Analgesia  (n=25) |
| --- | --- | --- |
|  |  |  |
| Preload dependency | 26 (62) | 16 (64) |

| **5^th^ postoperative day** | Epidural Analgesia  (n=14) | No Epidural Analgesia  (n=46) |
| --- | --- | --- |
|  |  |  |
| Preload dependency | 9 (64) | 33 (72) |

Numbers presented as n (%) *POD: postoperative day
